# Biochemical and Functional Analysis of Two *Plasmodium falciparum* Blood-Stage 6-Cys Proteins: P12 and P41

**DOI:** 10.1371/journal.pone.0041937

**Published:** 2012-07-27

**Authors:** Tana Taechalertpaisarn, Cecile Crosnier, S. Josefin Bartholdson, Anthony N. Hodder, Jenny Thompson, Leyla Y. Bustamante, Danny W. Wilson, Paul R. Sanders, Gavin J. Wright, Julian C. Rayner, Alan F. Cowman, Paul R. Gilson, Brendan S. Crabb

**Affiliations:** 1 Burnet Institute, Melbourne, Victoria, Australia; 2 Department of Medical Biology, The University of Melbourne, Victoria, Australia; 3 Cell Surface Signalling Laboratory, Wellcome Trust Sanger Institute, Cambridge, United Kingdom; 4 The Walter & Eliza Hall Institute of Medical Research, Parkville, Victoria, Australia; 5 Malaria Programme, Wellcome Trust Sanger Institute, Cambridge, United Kingdom; 6 Departments of Immunology and Medicine, Monash University, Victoria, Australia; 7 Department of Microbiology and Immunology, The University of Melbourne, Victoria, Australia; Bernhard Nocht Institute for Tropical Medicine, Germany

## Abstract

The genomes of *Plasmodium* parasites that cause malaria in humans, other primates, birds, and rodents all encode multiple 6-cys proteins. Distinct 6-cys protein family members reside on the surface at each extracellular life cycle stage and those on the surface of liver infective and sexual stages have been shown to play important roles in hepatocyte growth and fertilization respectively. However, 6-cys proteins associated with the blood-stage forms of the parasite have no known function. Here we investigate the biochemical nature and function of two blood-stage 6-cys proteins in *Plasmodium falciparum*, the most pathogenic species to afflict humans. We show that native P12 and P41 form a stable heterodimer on the infective merozoite surface and are secreted following invasion, but could find no evidence that this complex mediates erythrocyte-receptor binding. That P12 and P41 do not appear to have a major role as adhesins to erythrocyte receptors was supported by the observation that antisera to these proteins did not substantially inhibit erythrocyte invasion. To investigate other functional roles for these proteins their genes were successfully disrupted in *P. falciparum*, however P12 and P41 knockout parasites grew at normal rates *in vitro* and displayed no other obvious phenotypic changes. It now appears likely that these blood-stage 6-cys proteins operate as a pair and play redundant roles either in erythrocyte invasion or in host-immune interactions.

## Introduction

Malaria remains one of the most serious infectious diseases of humanity. The disease is caused by the infection and destruction of red blood cells and related sequelae by protozoan parasites belonging to the genus *Plasmodium*. Of the four major species that infect humans, *Plasmodium falciparum* and *P. vivax* are the most widespread with *P. falciparum* being the most pathogenic and responsible for an estimated 0.8–1.2 million deaths annually [1], [2]. Infants are particularly susceptible to the disease because of less developed immunity but if they survive repeated infections over many years, a degree of protective but non-sterilising immunity can be attained by several years of age. The development of immunity offers hope that vaccine based strategies might be used to reproduce or even generate superior levels of protection than natural infection. One family of proteins, the 6-cys domain proteins, are generating particular interest as vaccine candidates because of their presence on the surface of different life stages.

The 6-cys domain proteins are so called because they contain modules with six characteristic cysteines forming three intra-molecular disulphide bonds between C1 and C2, C3 and C6, and C4 and C5 [3]–[5]. There are at least nine members of the 6-cys family encoded in each of the several *Plasmodium* genomes sequenced to date that parasitise either primates, rodents or birds [6]–[9]. Most family members contain two 6-cys modules, but up to seven modules can be found in a single protein, in addition to incomplete modules containing fewer cysteine residues [6], [10]. About half of the 6-cys family members characterised to date possess glycosylphosphatidylinositol (GPI) moieties that anchor them to the outer leaflet of the plasma membrane, while those that lack GPI-anchors presumably remain associated with the parasite surface via interactions with other membrane proteins [8], [10], [11]. The first 6-cys protein discovered was cloned from a *P. falciparum* blood-stage antigen COS expression library and was termed P12 after its clone number [12]. We have subsequently shown that P12 is GPI-anchored, a blood-stage antigen, and is expressed on the merozoite [8], [13]. We also identified a second blood-stage 6-cys protein P41 and a third P38, that appears to be strongly expressed throughout the life-cycle [8]. P41 is not GPI-anchored and antibodies generated to the relatively long spacer region between its two 6-cys domains indicated surface expression by immunofluorescence microscopy [8]. P41 also could be a target of infected host humoral immune response since human malaria immune sera recognise the spacer region [8].

The first two 6-cys proteins for which antibodies were shown to inhibit progression through the lifecycle were P230 and P48/45. These proteins are expressed on the surface of gametes and antibodies to these inhibit the successful fusion of gametes in the mosquito gut [14]–[17]. Gene knockout studies subsequently showed that P48/45 and P230 were required by male gametes to efficiently fuse with female gametes [18], [19]. The knockout of sporozoite stage 6-cys proteins, P36 and P36p, inhibited progression to blood-stage infection and the phenotype could be enhanced by deleting both of the tandemly linked gene loci [20], [21]. Loss of these proteins caused the sporozoites to arrest during the hepatocyte growth stage, perhaps as a result of failure of knockout parasites to recognize hepatocytes, although the reason for growth arrest has not been settled [20], [21]. In the rodent malarial parasite *P. berghei*, the failure of Δ*p36* and Δ*p36p* sporozoites to progress to blood-stage infection serves to protect mice from subsequent challenge with wildtype parasites and thus dual knockout Δ*p36/*Δ*p36p* parasites, if generated in *P. falciparum*, could act as live attenuated vaccine [21]–[24].

In this report we investigate biochemical and functional aspects of the *P. falciparum* blood-stage expressed 6-cys proteins P12 and P41. We produced recombinant forms of P12 and P41 in both bacterial and mammalian expression systems and generated antibodies to these proteins for biochemical and functional studies. Using mammalian expressed and parasite derived proteins, interactions of the 6-cys with other proteins were examined and revealed P12 and P41 form a heterodimer. The potential functional role of P12 and P41 in erythrocyte invasion was explored by assaying native and recombinant proteins for erythrocyte binding activities, invasion-inhibition studies with antibodies, as well as by genetically disrupting their genes and assaying for growth defects.

## Materials and Methods

### Production of antibodies to recombinant P12 and P41 in *Escherichia coli*

From *P. falciparum* genomic DNA *p12* sequence corresponding to H26 through to S321 was amplified so that the N-terminal secretion signal sequence and C-terminal GPI-anchor signal sequence were excluded. The *p12* DNA fragment was ligated into the SacII and NcoI sites of pASK45(+) (ABI) in frame with a N-terminal Strep II tag and C-terminal 6×His tag. A fragment of *p41* excluding the N-terminal secretion signal sequence (K21 to S378), was similarly amplified and ligated into the pASK45(+) as per *p12*. After inducing the expression of the 6-cys proteins in *E. coli* with 0.2 mg/L anhydrotetracycline (as per manufacturer's instructions) the bacteria were harvested and their inclusion bodies containing insoluble recombinant proteins were isolated as per [25]. The inclusion bodies were solubilised in 8 M urea and the 6×His tagged proteins were purified over Ni^2+^-NTA agarose under denaturing conditions (Qiagen). The proteins were then greatly diluted and refolded in the presence of glutathione redox pair and were further purified by anion exchange chromatography. P12 and P41 polyclonal rabbit sera and P12 mouse monoclonal antibodies were produced at the Walter & Eliza Hall Institute Monoclonal Antibody Facility.

### Western blot analysis of recombinant proteins and parasites

Recombinant *E. coli* produced P12 and P41 proteins were fractionated by SDS-PAGE under non-reducing and reducing (100 mM dithiothreitol) conditions followed by transfer onto nitrocellulose membranes. These were probed with malaria immune human IgG (5 µg/mL) followed by sheep anti-human horseradish peroxidase (1/1000). Signal was detected on X-ray film (Fuji) in the presence of chemiluminescence substrate (Pierce).

*P. falciparum* parasites were harvested at various stages throughout the asexual blood-stage cell cycle. They were lysed in 0.09% saponin to remove excess hemoglobin and were then solubilised in 1% Triton X-100 in PBS with complete protease inhibitor cocktail (Roche). After removing insoluble material by centrifugation (18000× *g*) the parasite lysates were mixed with SDS sample buffer prior to SDS-PAGE under non-reducing and reducing conditions. After transfer onto nitrocellulose membrane parasite proteins were probed with rabbit anti-P12 and anti-P41 IgGs (10 µg/mL) followed by goat anti-rabbit IRDye™ 800 (1/5000, Rockland). Mouse monoclonals such as that for P12 (20 µg/mL) were detected with goat anti-mouse IRDye™ 700 (1/5000). The secondary antibodies were imaged with a LI-COR Odyssey FC instrument.

### Parasite culturing

*P. falciparum* strains 3D7 and CS2 were maintained in continuous culture as per [26]. To produce late stage protein extracts of parasites, infected erythrocytes (32–40 hrs post infection) were first isolated from uninfected erythrocytes by passage through magnetized columns (Miltenyi). The purified schizonts were then cultured at 37°C in RPMI media without Albumax until about half the schizonts had ruptured after which the remaining intact schizonts were pelleted by centrifugation (500× *g*) to produce 40–48 hr schizont extracts. The supernatant was then pelleted to enrich for the smaller merozoites (2000× *g*). To produce culture supernatant, the magnet-purified schizonts were incubated overnight until all had released merozoites that had subsequently shed their surface coats. The culture supernatant was clarified by pelleting the merozoites followed by concentration of the supernatant proteins through 10 kDa cut-off spin concentrators (Millipore).

### Immunofluorescence microscopy

Late schizonts and merozoites were fixed with 4% paraformaldehyde/0.0075% glutaraldehyde as described previously [27]. The cells were labelled with mouse and rabbit antibodies specific to MSP1 (4H9/19 [28]; 17b6), P12, and P41 as indicated and corresponding secondary antibodies Alexa Fluor 488 goat anti-rabbit IgG and Alexa Fluor 568 goat anti-mouse IgG (1/2000, Molecular Probes). The images were captured using Zeiss AxioObserver Z1 fluorescence microscope and analysed with ImageJ software.

### Mammalian expression and avidity-based extracellular interaction screening (AVEXIS)

To express P12 and P41 in a mammalian expression system, the regions of the genes encoding the predicted ectodomain fragments were chemically synthesized (Geneart AG, Germany) so that their codons were optimized for expression in human cells. In addition, any potential N-linked glycosylation sequons were mutated (N-X-S/T to N-X-A) to prevent inappropriate glycosylation and an exogenous signal peptide was used and cloned N-terminal to a rat Cd4 domain 3 and 4 (Cd4d3/4) tag using flanking NotI and AscI restriction sites as described [29], [30]. Proteins were produced as secreted 6×His tagged recombinant proteins using HEK293E cells and purified using Ni^2+^-NTA resin, essentially as described [31]. AVEXIS assays were performed as described [31].

### Size exclusion and surface plasmon resonance (SPR)

Binding of recombinant P12 and 41 (recP12 and recP41 respectively) to form a heterodimer was examined by column shift assay. Equimolar amounts of recP12-Cd4d3/4-6H (150 µg at 2.0 mg/mL) and recP41-Cd4d3/4-6H (180 µg at 3.5 mg/mL) were co-incubated for 1 hr at 37°C in phosphate buffer (containing 250 mM imidazole). Following incubation samples were centrifuged for 10 minutes at 18000× *g* at 4°C prior to filtration through 0.2 µm Acrodisc 13 mm Syringe Filters (Pall Corporation). This material underwent gel filtration chromatography using a Superdex 200 10/300 GL column (GE Healthcare) in PBS on an ÄKTA Purifier (GE Healthcare). recP12-Cd4d3/4-6H (300 µg) and recP41-Cd4d3/4-6H (360 µg) were also subjected to gel filtration (Superdex 200 10/300 GL) independently to determine their individual retention volumes as a point of comparison with the heterodimer.

Biophysical binding analysis was performed by surface plasmon resonance using a T100 instrument (BIAcore). Briefly, proteins were captured on streptavidin-coated sensor chips (Biacore, GE Healthcare) by a C-terminal enzymatically biotinylatable tag “bio” as described [31]. Approximately 150RU of biotinylated rat Cd4d3/4 (tag alone) was captured in the flow cell used as a reference and approximate molar equivalents of recP12-Cd4d3/4-bio or recP41-Cd4d3/4-bio immobilised in the other flow cell. Purified recP12-Cd4d3/4-6H or recP41-Cd4d3/4-6H proteins were resolved by gel filtration just prior to use in SPR experiments to remove small amounts of protein aggregates which are known to influence kinetic binding measurements. Increasing concentrations of purified proteins were injected at high flow rates (100 µL/min) to minimise rebinding effects for kinetic studies or at 10 µL/min for equilibrium analysis. Both kinetic and equilibrium binding data were analysed in the manufacturer's evaluation software. Equilibrium binding measurements were taken once equilibrium had been reached using reference-subtracted sensorgrams. All experiments were performed at 37°C.

### Immunoprecipitations

Rabbit pre-immune, anti-P12, and anti-P41 IgGs were firstly immobilised on Protein G agarose as per manufacturer's instructions (Pierce). Parasite lysates prepared as above were incubated with IgG-immobilised Protein G agarose overnight at 4°C. The unbound lysates were collected for further analysis and the resins were then washed with 1% Triton X-100 in PBS to remove unbound proteins. Bound proteins were eluted off the resins using 0.1 M glycine (pH 2.8) solution. The pH of the samples were adjusted to 7 using 1 M Tris-HCl buffer (pH 9.0) and were prepared in non-reducing sample buffer for western blot analysis. For protein identification by LC-MS/MS sequencing, samples were prepared in reducing sample buffer for SDS-PAGE. Protein gels were stained with GelCode Blue Stain reagent as per manufacturer's instructions (Pierce).

### Chemical crosslinking

Schizont-stage parasites were subjected to magnet purification as described earlier. Samples were divided into 5 equal aliquots and resuspended in 5 mL PBS with complete protease inhibitor cocktail (Roche). Dithiobis(succinimidylpropionate), DSP (Thermo), prepared in dimethyl sulfoxide (Sigma) was added to the final concentration of 0 µM, 62.5 µM, 125 µM, 250 µM, and 500 µM, and parasites were crosslinked for 30 minutes at room temperature. Parasite material was subsequently pelleted and crosslinking reactions were quenched for an additional 30 minutes at room temperature by the addition of 5 mL of quenching solution (25 mM Tris-HCl, 120 mM NaCl, pH 7.5). The crosslinked samples were washed and subsequently lysed by 0.09% saponin. They were then solubilised in 1% Triton X-100 in PBS and prepared for western blot analysis in non-reducing sample buffer.

### Erythrocyte binding assay and depletion assay

Erythrocyte binding assays were carried out as previously described with slight modifications [32]. Briefly, 250 µL of culture supernatant containing invasion ligands was mixed with 50 µL of packed fresh erythrocytes for 30–60 minutes at room temperature. The RBCs were separated from supernatant by spinning through 400 µL of dibutyl phthalate (Aldrich) at 12000× *g* for 30 seconds. Proteins bound to the erythrocytes were eluted by incubating with 10 µL of 1.6 M NaCl in PBS at room temperature for 10 minutes. Eluted proteins were obtained after 4 minutes of centrifugation at 12000× *g* and mixed with equal volume of 2× non-reducing sample buffer. The eluates were analysed by western blotting as described above. Depletion assays were also performed to determine the RBC-binding activity of P12/P41. Culture supernatant was serially incubated five times with batches of uninfected erythrocytes at 20% hematocrit. Each incubation was performed at room temperature for an hour. Aliquots of supernatant from each round were analysed by western blotting.

### Double crossover gene knockouts

To generate knockout constructs for 3D7 and CS2 parasites, 5′ and 3′ sequences of *p12* and *p41* genes were amplified from parasite genomic DNA and cloned into pCC1 plasmid [33]. The 5′ flanks of *p12* and *p41* were cloned into the plasmid via SacII/SpeI restriction sites. The 3′ flanks of both genes were cloned into the pCC1 plasmid via EcoRI/AvrII sites. Primers used to generate the knockout plasmids are described in supplementary information (Table S1). These knockout plasmids were transfected into 3D7 and CS2 parasites and the double crossover knockout parasites were selected as outlined in [33]. Clonal lines of knockout parasites were achieved by limiting dilution.

### Southern blot and PCR analyses

Genomic DNA of knockout parasites were digested with restriction enzymes, XbaI/AvaII for Δ*p12* parasites and HindIII/SphI for Δ*p41* parasites, and transferred to a nylon membrane after electrophoresis. The membrane was probed with dioxygenin (DIG)-labelled 5′ flank and 3′ flank, amplified as described above, followed by chemiluminescent detection per manufacturer's instructions (Roche). Specific primers were also designed to confirm homologous recombination in knockout parasites by PCR (supplementary information; Table S2).

### Growth measurement and invasion inhibition assay

Synchronous cultures of 3D7, Δ*p12*, Δ*p41*, and Δ*p36* parasites were adjusted to 1% parasitemia and were subsequently diluted 100,000 fold in duplicate. They were continuously cultured for 6 cycles, when one of the parasite lines grew back up to approximately 1% parasitemia, and all parasites were counted and calculated for the amplification rates. One-way ANOVA analysis was performed using Prism software (GraphPad).

The invasion inhibition assay was performed as previously described [34]. Briefly, filtered purified merozoites were mixed with uninfected RBCs at appropriate parasitemia and hematocrit. Prepared cultures were added to a 96 well U-bottom plate in the presence of purified rabbit anti-P12 and anti-P41 IgGs made to *E. coli* recombinant proteins at 2 mg/mL final concentration. Non-immune rabbit IgG was used as a non-inhibitory control. All samples were run in duplicate. The 96-well plates were placed in humidified gassed chamber as per standard culture condition for an hour and the cultures were then washed twice with culture medium to remove the antibodies. The samples were prepared for flow cytometry analysis 40 hours later by resuspension in 100 µL of PBS with 10 µg/mL ethidium bromide (EtBr, Bio-Rad). They were stained for one hour prior to centrifugation, removal of the supernatant and resuspension in 200 µL of PBS. Parasitemia was measured on a Becton Dickinson FACSCalibur flow cytometer using a 488 nm laser for excitation of EtBr stained (Fl–2) parasites. Samples were analysed using FlowJo software (Tree Star Inc.) by first gating for intact erythrocytes with side scatter and forward scatter parameters, and subsequently determining the positive cells in FL2 channel. Invasion inhibition by the anti-P12 and anti-P41 antibodies was calculated as percentages of pre-immune rabbit IgG.

Invasion inhibition assays using rabbit polyclonal anti-P12 and anti-P41 IgGs generated against the HEK293E expressed proteins were performed as described in [35].

## Results

### Expression of recombinant P12 and P41 in *E. coli* and HEK293E mammalian cells

**Figure 1:**
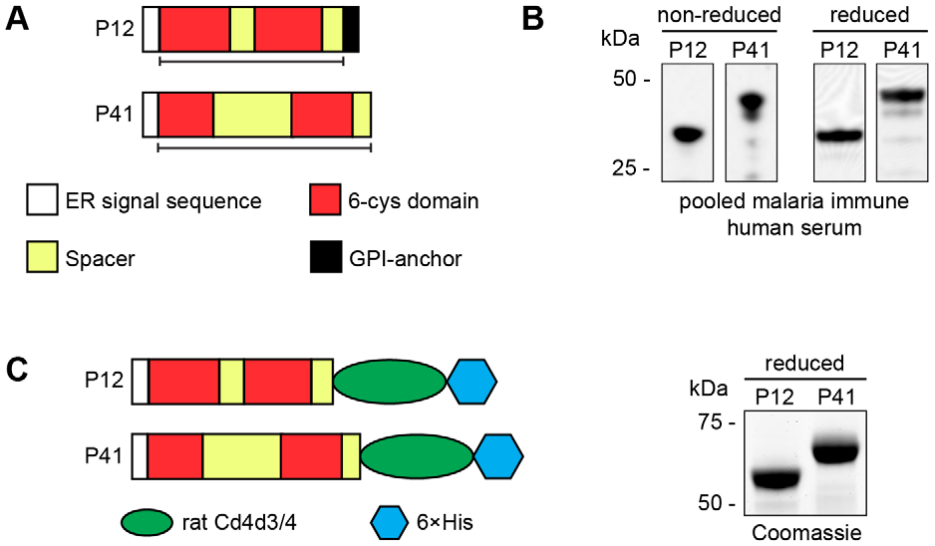
Recombinant P12 and P41 fusion proteins produced in *Escherichia coli* and HEK293E cell expression system. (A) Diagram of P12 and P41 showing regions (black lines) that were expressed in *E. coli*. Secretion signal sequences are in white, protein spacer regions in yellow, 6-cys domains are in red and the glycosylphosphatidyl inositol signal sequence is in black. (B) A western blot of recombinant reduced and non-reduced P12 and P41 expressed in *E. coli* probed with pooled malaria immune human serum. Molecular mass protein markers are indicated on the left. (C) P12 and P41 expressed recombinantly C-terminally fused to domain 3 and 4 of rat Cd4 (green) followed by 6×His (blue) (left) in HEK293E cells. Recombinant proteins were expressed as soluble proteins of expected sizes (Coomassie-stained gel, right).

A *p12* DNA fragment amplified from *P. falciparum* genomic DNA, excluding the N-terminal secretion signal sequence and C-terminal GPI-anchor signal sequence (H26 to S321, Fig. 1A), was expressed in *E. coli* to produce a fusion protein with an N-terminal Strep II tag and C-terminal 6×His tag. A fragment of P41, excluding the N-terminal secretion signal sequence (K21 to S378, Fig. 1A), was similarly expressed. Inclusion bodies containing insoluble fusion proteins were isolated from *E. coli* and were solubilised in 8 M urea and the fusion proteins were purified over Ni^2+^-NTA agarose under denaturing conditions. The proteins were then highly diluted and refolded in the presence of glutathione redox pair and were further purified by anion exchange chromatography. Equal amounts of the recombinant 6-cys proteins were fractionated by SDS-PAGE under reducing and non-reducing conditions. Western blots of these were probed with pooled malaria immune human serum and for both P12 and P41 bands of the expected size were detected (37 and 45 kDa, respectively, Fig. 1B). The P12 and P41 proteins were then used to immunise rabbits to produce polyclonal antibodies. Mice were also immunised with both proteins, however a monoclonal antibody was only obtained to P12.

P12 and P41 C-terminally tagged with rat Cd4d3/4 followed by 6×His were expressed in HEK293E mammalian cells (Fig. 1C; left panel). The purified recombinant P12 and P41 were soluble, migrated at the expected sizes by SDS-PAGE and were highly pure (Fig. 1C; right panel). Rabbit polyclonal antibodies were also raised against these mammalian recombinant P12 and P41.

### P12 and P41 are expressed in schizont-stage parasites and are shed into the culture supernatant

**Figure 2:**
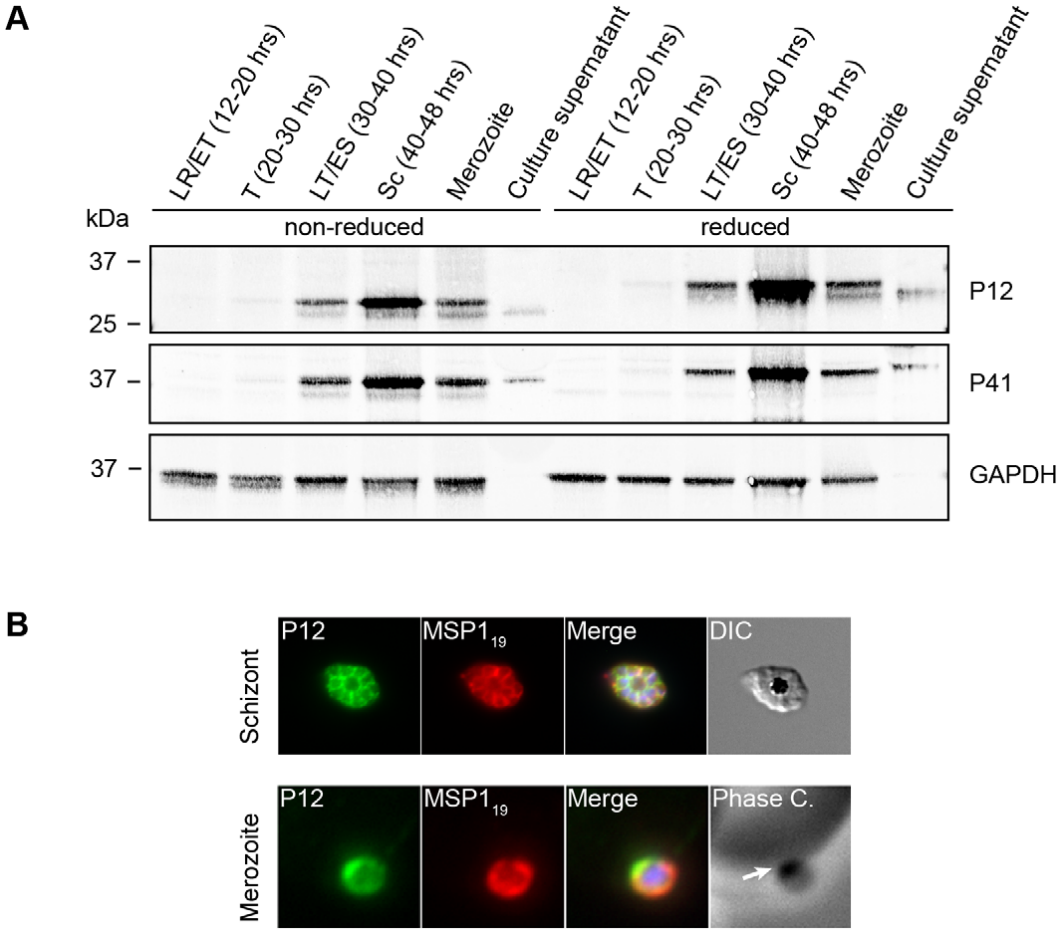
P12 and P41 are expressed in schizont-stage parasites and are shed into the culture supernatant. (A) Equal amounts of total protein from parasites harvested at different times throughout the asexual blood-stage cell cycle were subjected to SDS-PAGE in non-reducing and reducing conditions. Blots of these proteins were probed with rabbit IgGs raised to recombinant P12 and P41 (10 µg/mL) expressed in *E. coli* and demonstrated maximum expression of P12 and P41 in late stage parasites. Blots were also probed with rabbit anti-GAPDH (kindly supplied by Professor Leann Tilley) to ensure equal loading of parasite material from the various time points. (LR = late ring, ET = early trophozoite, T = trophozoite, ES = early schizont, Sc = schizont) (B) Localisation of P12 was explored by immunofluorescence assays. Schizonts and merozoites were fixed and labelled with mouse anti-MSP1_19_ (10 µg/mL) and rabbit IgG to P12 (50 µg/mL) followed by Alexa Fluor 488 goat anti-rabbit and Alexa Fluor 568 goat anti-mouse secondary antibodies IgG. P12 was found to localise to the parasitophorous vacuole in schizonts and on the merozoite surface upon comparison with MSP1_19_. Interestingly, P12 exhibited concentrated apical localisation on the merozoite surface (white arrow) similar to the published localisation of P41 [8]. The representatives of schizonts and merozoites are shown.

The antibodies raised to *E. coli* expressed P12 and P41 detected proteins of the expected size in the western blots of parasite proteins (Fig. 2A). The non-reduced parasite proteins showed greater mobility than reduced proteins attesting to the disulphide bonding in the former. Interestingly, the difference in mobility was not as pronounced in recombinant versions of P12 and P41 and whether this reflects improper folding is unknown. However, the proteins appear to have generated effective antibodies. Parasite proteins with invasion-related functions are usually made late in the 48 hr cell cycle and become incorporated into developing merozoites. To ascertain if this was the case with P12 and P41, parasites were harvested at 8–10 hour intervals post invasion and extracted proteins were subjected to SDS-PAGE and western blotting under reducing and non-reducing conditions. P12 and P41 were first detected in the trophozoite stage, 30–40 hrs post invasion (HPI) and the proteins reached their peak expression in schizont stage, 40–48 HPI (Fig. 2A). Both proteins were faintly detected in 12–20 and 20–30 HPI stage parasites, though this may have been due to low level contamination with older parasites since the published transcriptome data for *p12* and *p41* does not indicate transcription at this time [36], [37]. Moreover, P12 and P41 were also detected in culture supernatant suggesting that these proteins are shed from the merozoite surface (Fig. 2A). We note that P41 and GAPDH migrate as a single band in reduced samples and as two bands in non-reduced samples, and consider the latter to be caused by the electrophoretic conditions rather than extra post-translational processing. Of note, two forms of P12 were present in parasite samples under both reducing and non-reducing conditions while in culture supernatant only the smaller form was detected (Fig. 2A). This likely indicates a form of proteolytic cleavage is responsible for the shedding of P12 from the merozoite surface, possibly occurring in near proximity to the GPI-anchor, as similarly occurs for merozoite surface protein 1 (MSP1) [38]. The protease subtilisin 2 is known to cleave several proteins from the merozoite surface and is therefore possibly responsible for P12 cleavage [38]. In the immature parasite samples a degree of proteolytic cleavage of P12 appears to have occurred during parasite lysate preparation, despite the inclusion of protease inhibitors (Fig. 2A).

### P12 localises to the merozoite surface

We have previously shown that P41 resides in the parasitophorous vacuole space of the segmented schizont and on the merozoite surface by immunolabelling. GFP-tagging of P12 indicates that it is also surface localised [8]. However, the localisation of native P12 has not been previously confirmed given that GFP-tagging can result in incorrect trafficking [39]. Rabbit P12 anti-serum was employed here to probe the location of the native protein. Schizonts and ruptured merozoites adhering to the surface of erythrocytes were examined since although they do not appear to be invading, are still likely to retain their adhesive surface coats. We have found that native P12 is located in the parasitophorous vacuole space of segmented schizonts and it is present on the surface of merozoites (Fig. 2B). In addition, P12 shows an increased concentration towards the darkly contrasting apical region visualized by phase contrast microscopy, which is different from the established surface marker MSP1 (Fig. 2B, white arrows).

### Recombinant P12 and P41 form a stable heterodimer

**Figure 3:**
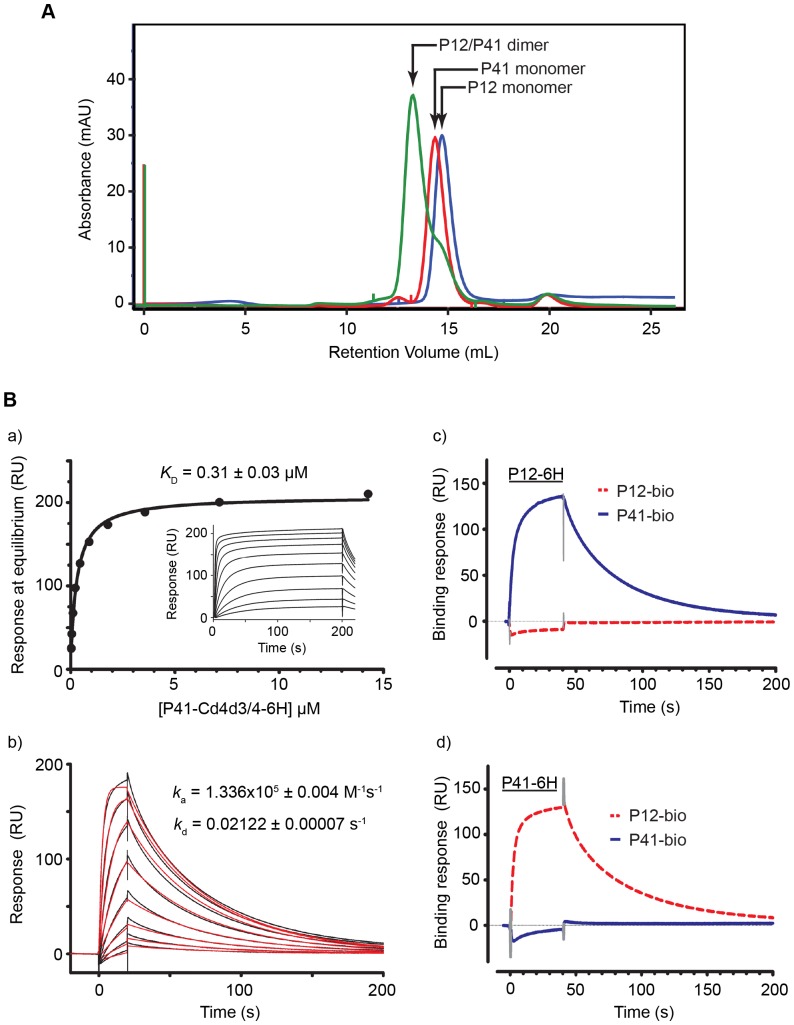
Recombinant P12 and P41 form a heterodimer. (A) The recP12-Cd4d3/4-6H (blue line), recP41-Cd4d3/4-6H (red line) and the co-incubated proteins (green line) were resolved on a Superdex 200 10/300 GL column. Upon co-incubation, a single peak was observed at a lower retention volume than that observed for the individual proteins, corresponding in size to a P12/P41 heterodimer. (B) Biochemical analysis of the recombinant P12/P41 interaction by surface plasmon resonance. (a) Equilibrium dissociation constant (*K*_D_) for P12/P41 interaction. Serial dilutions of recP41-Cd4d3/4-6H were injected through flow cells containing Cd4d3/4 (as a reference) and recP12-Cd4d3/4-bio captured on a streptavidin-coated sensor chip until equilibrium had been reached (see inset). Reference-subtracted binding data were plotted as a binding curve and an equilibrium dissociation constant calculated using non-linear regression fitting of a simple Langmuir binding isotherm to the data (solid line). (b) Kinetic data for the P12/P41 interaction. Serial two-fold dilutions of 3.6 µM purified recP41-Cd4d3/4-6H were injected at high flow rates (100 µL min^−1^) over recP12 and a reference flow cell. Sensorgrams showed excellent fits to a simple 1∶1 binding model (shown in red) and were used to derive association (*k*_a_) and dissociation (*k*_d_) rate constants. (c, d) P12 and P41 do not interact homophilically. (c) Injection (solid bar) of purified 2.5 µM recP12-CD4d3/4-6H over recP41-Cd4d3/4-bio (blue line) and recP12-Cd4d3/4-bio (red dotted line) immobilised on a streptavidin-coated sensor chip. The reciprocal experiment using purified P41 is shown in (d).

Because P41 does not possess a GPI-anchoring sequence or predicted transmembrane domain, this raises the possibility that P41 interacts with another protein(s) on the merozoite surface. Since the GPI-anchored P12 shares the same expression profile and localisation as P41, we hypothesised that they may be interacting partners as has been reported for other 6-cys family members, P230 and P48/45, on the gametocyte surface [11]. Heterodimerisation of recP12-Cd4d3/4-6H and recP41-Cd4d3/4-6H was first established by means of a column shift assay following co-incubation in PBS at 37°C for an hour. The retention volume of the co-incubated 6-cys proteins was compared to that of the individual proteins by gel filtration chromatography. The co-incubated proteins eluted as a major peak at a retention volume of 13.2 mL and a less abundant shoulder possibly representing monomeric protein (Fig. 3A, green line). The individual proteins were resolved with retention volumes of 14.69 mL (recP12-Cd4d3/4-6H) and 14.34 mL (recP41-Cd4d3/4-6H). When plotted on a curve of molecular weight standards (linear log MW curve), the elution volumes of recP12-Cd4d3/4-6H and recP41-Cd4d3/4-6H corresponded to proteins with an apparent molecular weight of ∼90 kDa and ∼105 kDa respectively. The major peak resolved for co-incubated material eluted at a retention volume corresponding to an apparent molecular weight of ∼180 kDa indicative of heterodimer formation. It is worthy to note that estimation of molecular weight by gel filtration is confounded by factors including buffer type, interactions with the chromatographic medium, particle size distribution and ultimately only fractionates proteins on the basis of their Stokes radius, not molecular weight.

Surface plasmon resonance was then used to characterise the physical properties of the recP12-Cd4d3/4-6H and recP41-Cd4d3/4-6H interaction, and established an equilibrium dissociation constant (*K*_D_) for the complex of 0.31±0.03 µM (Fig. 3B; a). We also measured the kinetic properties of the interaction which was calculated as 1.336×10^5^±0.004 M^−1^ s^−1^ for the association rate constant (*k*_a_) and 0.02122±0.00007 s^−1^ for the dissociation rate constant (*k*_d_) (Fig. 3B; b). To determine whether P12 and P41 could interact homophilically, we independently injected purified recP12-Cd4d3/4-6H or recP41-Cd4d3/4-6H over the same proteins that had been immobilised using a C-terminal biotin tag on streptavidin-coated sensor chips. When 2.5 µM of recP12-Cd4d3/4-6H (solid bar) was injected over the chip, an interaction was observed with recP41-Cd4d3/4-bio (blue line), but not recP12-Cd4d3/4-bio (red dotted line) (Fig. 3B; c). The reciprocal experiment, where 2.5 µM of recP41-Cd4d3/4-6H was injected, confirmed the interaction between the hetero-species (Fig. 3B, d). There was no evidence that either recP12 or recP41 are able to interact with themselves in a homophilic interaction.

### P12 and P41 form a heterodimer on the merozoite surface

**Figure 4:**
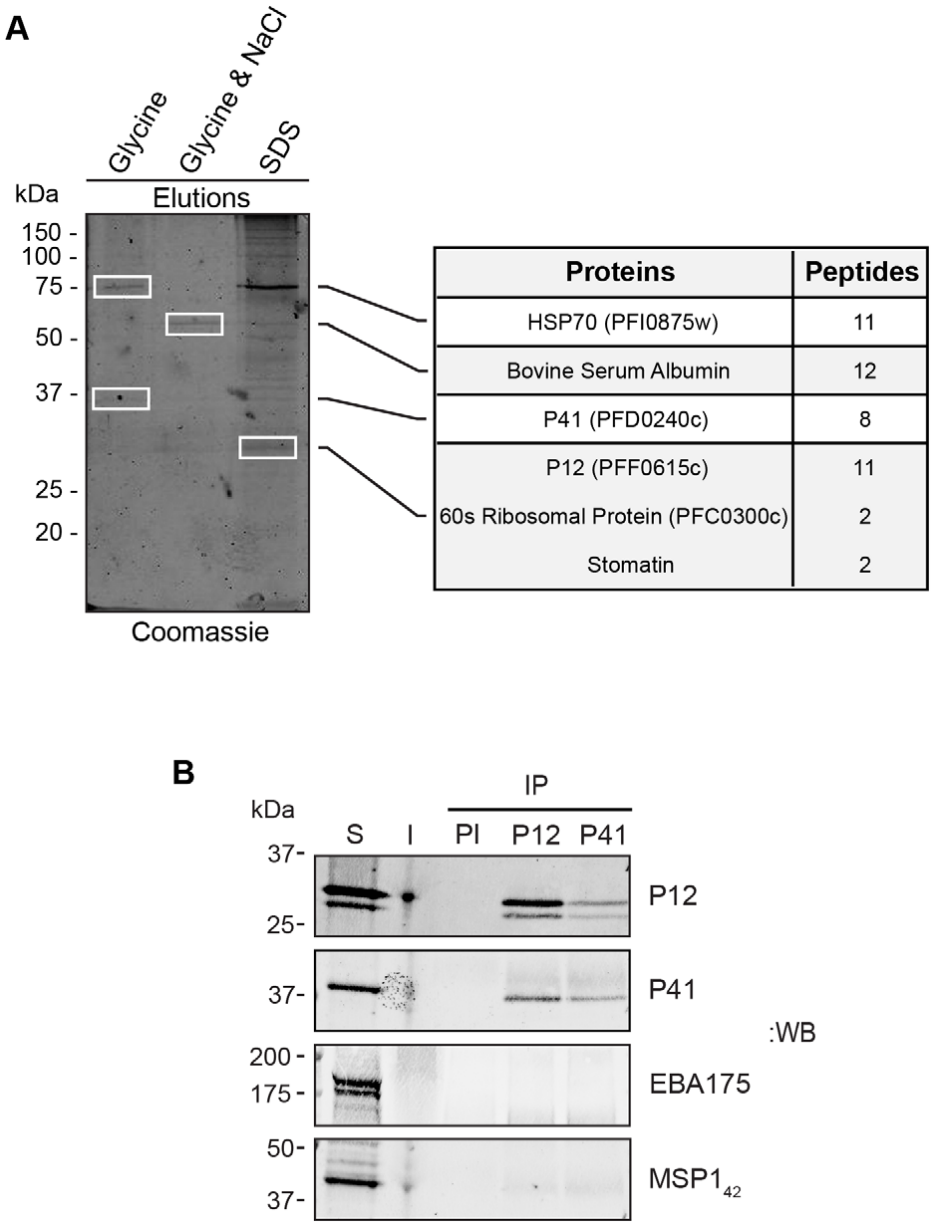
Native P12 and P41 interact as a heterodimer. (A) A schizont lysate of 3D7 parasites in 1% Triton X-100 in PBS was subjected to immunoprecipitation using rabbit anti-P12 IgG crosslinked to Protein G agarose. Bound proteins were serially eluted off the beads by 0.1 M glycine, 0.1 M glycine with 1 M NaCl, and 2% SDS. Eluates, along with the unbound material were analysed by SDS-PAGE followed by colloidal Coomassie staining. Proteins immunoprecipitated by anti-P12 IgG were excised from the polyacrylamide gel and evaluated by LC-MS/MS sequencing. P41, HSP70, 60 s ribosomal protein, and BSA were identified, however given its co-elution in glycine, P41 was the most likely binding partner of P12. (B) The P12 and P41 interaction was also confirmed by western blotting of immunoprecipitated material using pre-immune (PI), anti-P12, and anti-P41 rabbit IgG coupled Protein G agarose. The 1% Triton X-100 soluble (S) and insoluble (I) parasite extracts were included as controls. Blots were also probed with anti-EB175 and anti-MSP1_19_ sera to demonstrate the specificity of the P12/P41 interaction.

Having established heterodimerisation of recombinant P12 and P41, we investigated whether this held true for the native proteins by performing co-immunoprecipitation experiments with 6-cys antibodies crosslinked to Protein G agrose resin. Immunoprecipitations were performed on 3D7 schizont lysates using rabbit anti-P12 IgG. Eluted precipitates were fractionated by SDS-PAGE and stained with colloidal Coomassie. The most strongly staining bands were excised from the gel and proteins identified by LC-MS/MS sequencing (Fig. 4A). In addition to a band of ∼32 kDa corresponding to P12, a second larger species which co-eluted in low pH glycine was identified as P41 indicating P12 and P41 are likely to be associated in late stage parasites. Other species eluted under more stringent conditions were identified by LC-MS/MS, but proved to contain abundant parasite proteins not derived from the merozoite surface (e.g. ER localised HSP70 BIP), and are therefore likely to represent non-specific interactions (Fig. 4A). P41 could also be clearly detected in western blots of anti-P12 immunoprecipitates (Fig. 4B). Reciprocal experiments immunoprecipitating P41 also co-purified P12, but other surface proteins such as EBA175 and MSP1 could not be detected (Fig. 4B). Further immunoprecipitation experiments identified no other specific interacting partners (data not shown). We therefore conclude that native P12 and P41 are interacting partners.

To establish whether the native P12/P41 complex represents a single heterodimer or a higher order complex, we stabilized the complex with a reduction-sensitive chemical crosslinker DSP. Crosslinking demonstrated a single P12 and P41 heterodimer, since no larger complex formation was observed (Fig. S1). We also determined that, in the absence of P41, P12 does not homo-dimerize in the parasite, consistent with the gel filtration and surface plasmon resonance studies (Fig. S1). The crosslinked samples also served to explore potential weak or transiently interacting partners of the P12/P41 dimer that could not remain associated under conventional immunoprecipitation conditions. While MSP1, MSP9, and SERA5 were identified as associating with the P12/P41 dimer, we consider this interaction is likely a consequence of random crosslinking possibly due to dense surface packing of these proteins (Fig. S2).

### Native P12 and P41 from culture supernatants do not bind to erythrocytes

**Figure 5:**
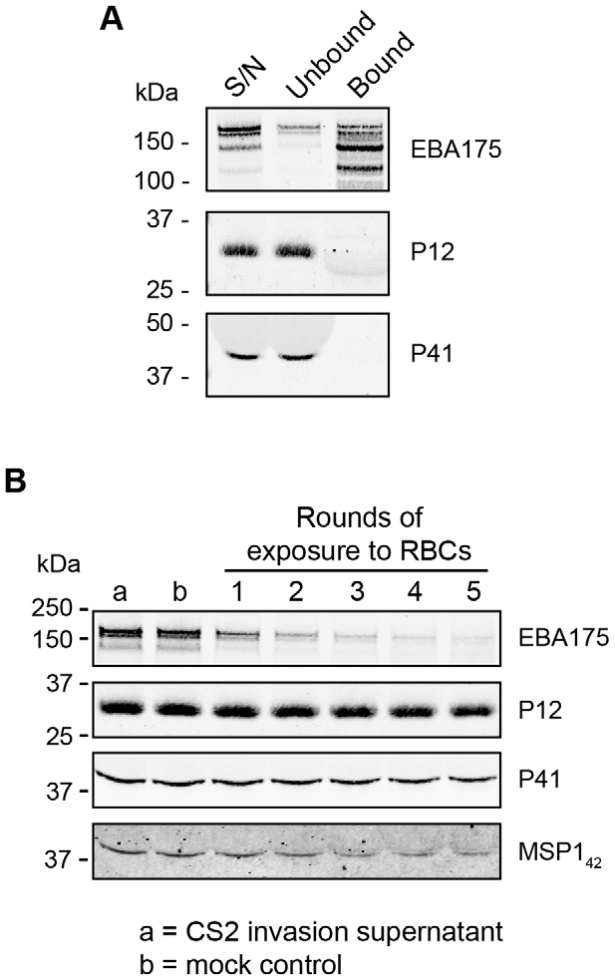
Native P12 and P41 from culture supernatant do not bind to erythrocytes. (A) Invasion supernatant (S/N) containing P12 and P41 was incubated with uninfected erythrocytes and bound proteins were eluted from the erythrocytes once centrifuged through oil. EBA175, a known erythrocyte binding protein, bound to the erythrocytes as expected; however, P12 and P41 did not. (B) Lack of binding of P12 and P41 to erythrocytes was also confirmed via an erythrocyte binding protein depletion assay to test for weak interactions between P12/P41 and RBCs. The relative amounts of target protein in starting material and in mock incubation (i.e. no erythrocytes) are shown in lanes marked a & b, respectively.

Since both P12 and P41 were localised to the surface of the merozoite, we investigated whether they serve as a parasite invasion ligand binding to an erythrocyte receptor in the same manner as other merozoite surface proteins such as EBA175. Culture supernatant containing native P12 and P41 was used in preference to recombinant proteins in the event that a functional binding complex is dependent upon additional interacting proteins that remain unidentified. Culture supernatant was incubated with uninfected erythrocytes and cells were centrifuged through oil (dibutyl phthalate), washed, and eluted with a high salt solution as has previously been performed successfully for other ligands [40]. Western blot analysis however detected no binding for P12 and P41 in contrast to EBA175, a known invasion ligand that binds to Glycophorin A (Fig. 5A).

The oil-based erythrocyte binding assay is considered of reasonable stringency given the multiple washing steps to remove weakly interacting proteins. Consequently a second less stringent approach was adopted whereby aliquots of uninfected erythrocytes were serially incubated with a single sample of culture supernatant to deplete its erythrocyte binding proteins. Upon comparison to the starting material and the mock incubated material (a,b respectively, Fig. 5B) each round of erythrocyte incubation did not substantially deplete P12 and P41. As a positive control, EBA175 was significantly depleted to <10% of its original amount following 5 rounds of erythrocyte incubation (Fig. 5B). The comparatively weaker-binding MSP1-42 fragment of MSP1, which has been demonstrated to bind to heparin sulphate-like sugars on the erythrocyte surface, was also depleted to ∼30% of its original amount [41]. While a slight depletion of P12 and P41 following 5 rounds of exposure to fresh erythrocytes may be due to extremely weak interactions with the erythrocyte surface, it is more likely attributable to small dilution effects at each binding step. We therefore conclude that since the heterodimeric form of P12/P41 shed from the merozoite surface does not appear to bind fresh erythrocytes the complex probably does not have a direct erythrocyte-binding role during invasion.

### P12 and P41 are not essential for blood-stage development

**Figure 6:**
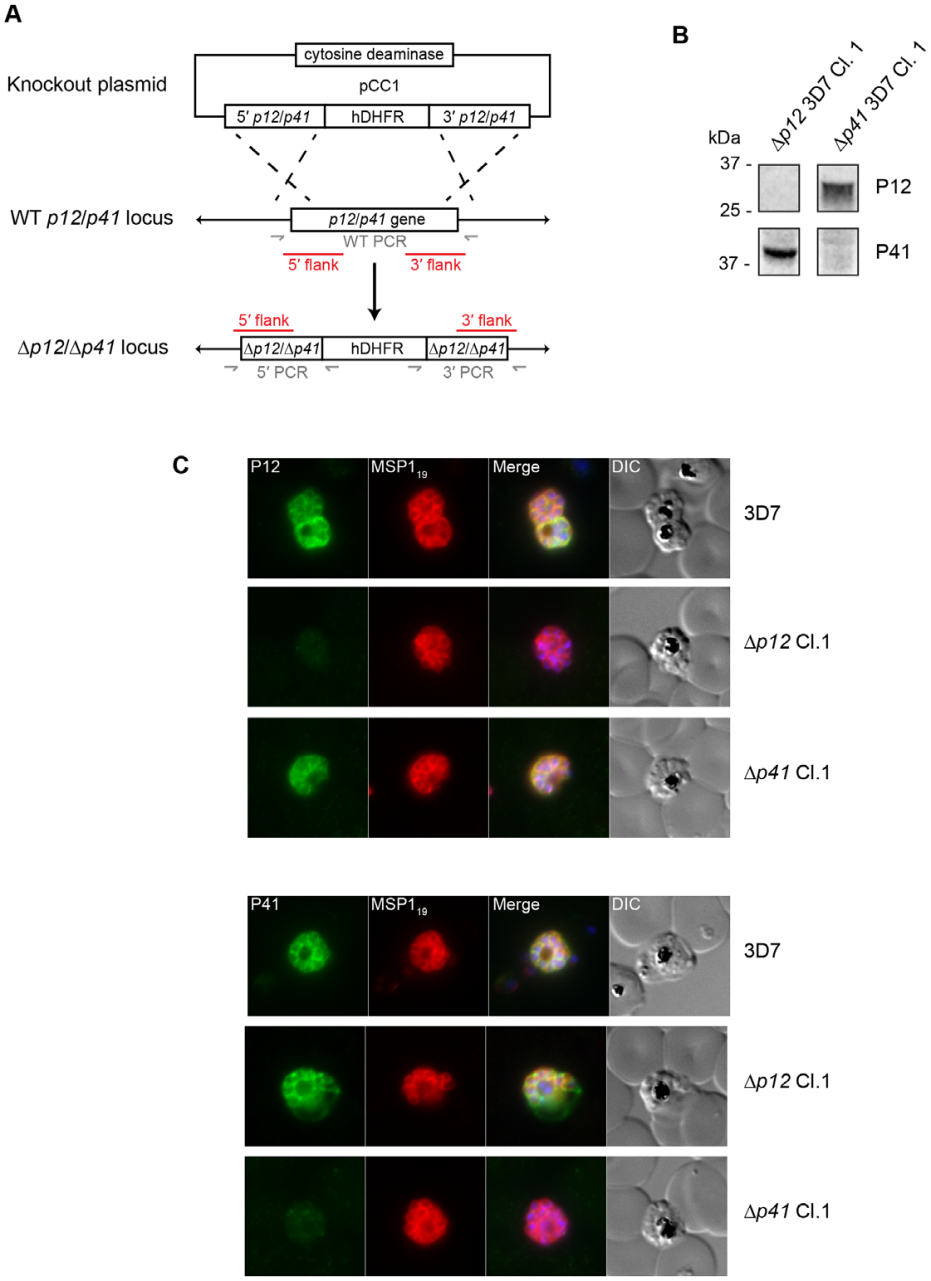
The genes for P12 and P41 can be disrupted and are therefore not essential for parasite growth. (A) Diagram of plasmid and strategy for disrupting *p12* and *p41*. A positive drug selection gene encoding a human dihydrofolate reductase (hDFHR) gene is flanked by sequences derived from the P12/P41 genes that will facilitate integration into the locus by double homologous recombination. To eliminate parasites in which integration has not occurred, a negative drug selection cytosine deaminase gene is included to remove the plasmid backbone from the transfected parasite population. (B) Western blots indicate that Δ*p12* and Δ*p41* lines do not express proteins corresponding to their respective disrupted genes. (C) Immunofluorescence microscopy of Δ*p12* and Δ*p41* and parental 3D7 parasites probed with rabbit anti-P12 and anti-P41 IgGs in addition to the merozoite surface marker MSP1 mAb indicate absence of protein expression in the mutants aside from minor cross-reactivity.

Different subsets of 6-cys proteins are expressed at different stages of the parasite life cycle, and deletion of the genes encoding for 6-cys proteins in sexual and liver stages resulted in dramatic phenotypes. This suggests a broad role for this family of proteins in regulating parasite development [18]–[21], [23], [24]. To test the hypothesis that P12 and P41 perform a similar function in blood-stage parasites, we performed *p12* and *p41* genetic deletions using a homologous recombination strategy outlined in Fig. 6A. The mutagenesis strategy involved using a positive drug selection gene (hDHFR) to produce transfected parasite lines carrying the integration plasmid and then a negative drug gene (cytosine deaminase) favouring the removal of the plasmid backbone and the disruption of the coding sequences of *p12* and *p41* by insertion of the positive drug selection cassette via double recombination [33]. Laboratory parasite lines 3D7 and CS2 were transfected with the gene-knockout plasmids and following positive/negative drug selection, gene replacement of the *p12* and *p41* loci were confirmed by Southern blot analysis (Fig. 6A, Fig. S3A). Southern blots indicated the Δ*p41* parasites were of a mixed population including wildtype parasites therefore necessitating the generation of clonal lines by limiting dilution. Genomic DNA (gDNA) was then extracted from several of the clones and a PCR was used to confirm in which lines deletion of *p12* and *p41* had occurred. This strategy involved amplification across both 5′ and 3′ crossover regions with specific primers (Fig. 6A, Fig. S3B). Protein extracts from Δ*p12* and Δ*p41* parasites were then prepared for SDS-PAGE and western blotting confirming the selected mutant lines no longer expressed their respective 6-cys proteins (Fig. 6B). Likewise, immunofluorescence microscopy of mutant schizont stage parasites confirmed loss of protein expression in vacuole space surrounding the developing merozoites (Fig. 6C).

### Δ*p12* and Δ*p41* parasites did not display altered growth or invasion phenotypes

**Figure 7:**
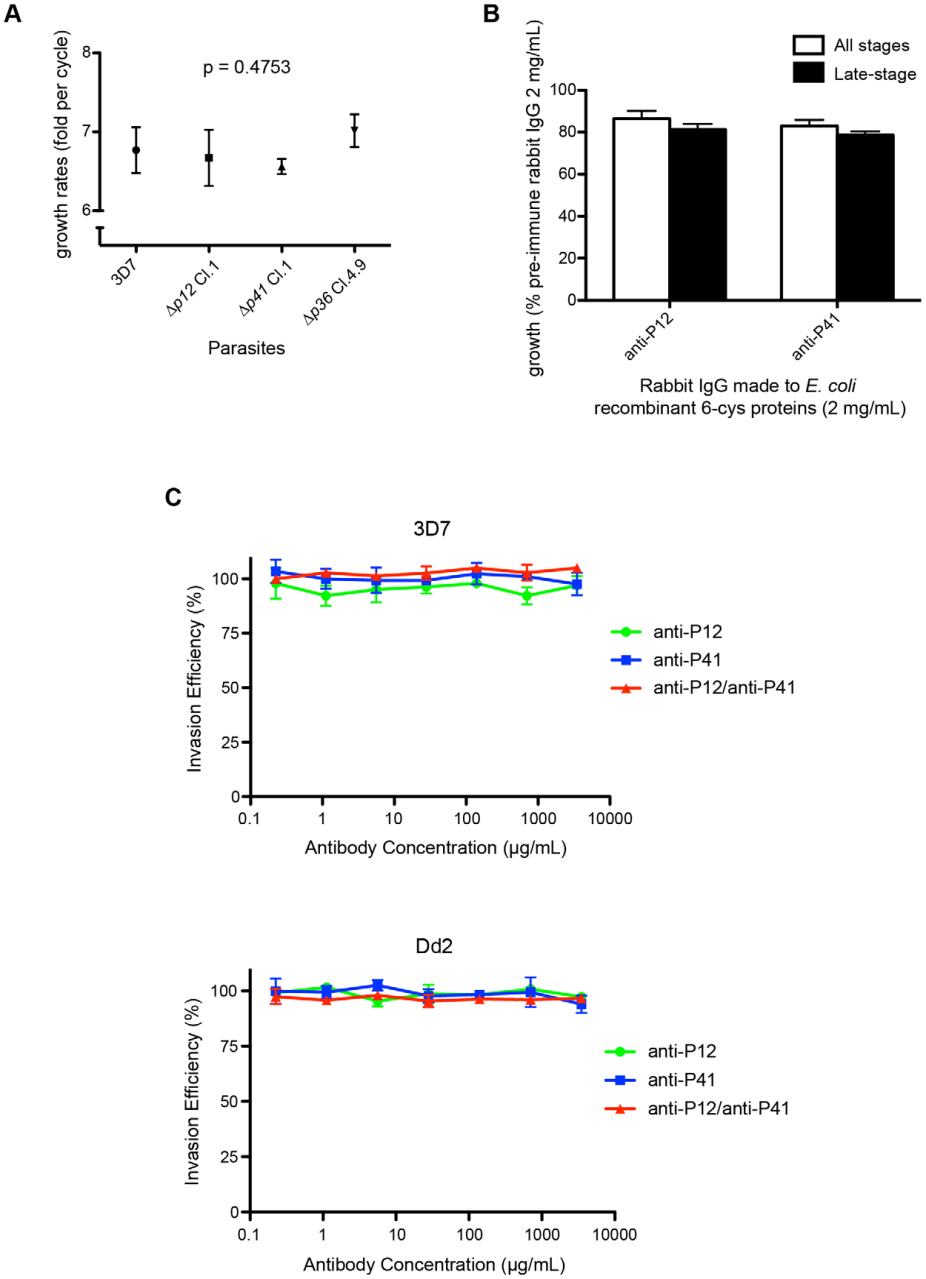
Genetic knockout growth analysis and invasion inhibition assays suggest a non-essential role for P12/P41 *in vitro*. (A) Growth rates of Δ*p12* and Δ*p41* mutants were measured. All parasite lines, 3D7, Δ*p12* clone 1, Δ*p41* clone 1, and Δ*p36* clone 4.9, were adjusted to 1% parasitemia and were subsequently diluted 100,000 fold in duplicate. Parasites were cultured for 6 cycles, until 1% parasitemia was achieved for one of the four parasite lines, then parasites were counted to calculate their rate of amplification. As shown in the graph (mean with SD), 3D7, Δ*p12* clone 1, Δ*p41* clone 1, and Δ*p36* clone 4.9 had similar growth rates of 6.77, 6.67, 6.56, and 7.02 fold per cycle, respectively, which is not statistically different (p = 0.4753, one-way ANOVA analysis). (B) Invasion inhibition assay indicated that antibodies to *E. coli* recombinant P12 and P41 only weakly inhibited merozoite invasion. Purified merozoites were incubated with rabbit polyclonal anti-P12 or anti-P41 IgGs at the final concentration of 2 mg/mL in the presence of uninfected erythrocytes. New infections were measured by counting parasitemia 40 hrs later by flow cytometry. Using rabbit pre-immune IgG as a control, the results indicated that anti-P12 and anti-P41 antibodies could inhibit parasite invasion by 15–20%. Total parasitemias and schizont-stage parasite populations were counted. (C) Invasion inhibition assays using antibodies generated against the HEK293E expressed P12 and P41 failed to demonstrate invasion inhibitory activity. Late trophozoite stage parasites were incubated with rabbit polyclonal anti-P12 or anti-P41 antibodies either independently or in combination. Final concentrations are shown. Parasites were cultured for 24 hours, and re-invasion was measured by flow cytometry. All assays were performed in triplicate, and invasion efficiency calculated comparatively with parasites incubated in the absence of antibodies. No significant difference in invasion efficiency was observed at any concentration of IgG.

Analysis of Δ*p12* and Δ*p41* parasite lines established that neither gene is essential for blood-stage growth. However, other possible phenotypes such as reduced growth rate due to less efficient merozoite maturation and invasion were investigated. We therefore compared the growth rates of the Δ*p12* and Δ*p41* parasites with the similarly constructed Δ*p36* parasite line that includes the same hDHFR selection marker [24]. P36, also a 6-cys protein, is exclusively expressed in sporozoites and is therefore highly unlikely to have a role in blood-stage growth. All three deletion lines, made in a 3D7 strain background, were adjusted to 1% parasitemia, diluted 100,000 fold and grown until the fastest growing line had amplified again to ∼1%. Parasitemias of all strains were then measured and amplification rates per cell cycle were calculated (Fig. 7A). 3D7, Δ*p12* clone 1, Δ*p41* clone 1, and Δ*p36* clone 4.9 parasites had growth rates of 6.77±0.29, 6.67±0.36, 6.56±0.10, and 7.02±0.21 fold per cell cycle, respectively. Parasite lines were subjected to one-way ANOVA analysis which showed no significant difference in growth rate across all lines.

### P12 and P41 antisera do not substantially inhibit parasite growth

It has been established that invasion ligands do not necessarily require direct binding to erythrocyte receptors in order to have an invasion related function. Such is the case for AMA1, which binds to the RON complex injected into the erythrocyte surface by the merozoite [42], [43]. The AMA1-RON complex forms part of the tight junction through which the parasite moves to invade its new host cell [42], [43]. Since antibodies raised to AMA1 are some of the most potent inhibitors of *P. falciparum* invasion known, we decided to test invasion inhibition by anti-P12 and anti-P41 IgGs, made to *E. coli* recombinant proteins. Relative to pre-immune control IgG, anti-P12 and anti-P41 IgGs only demonstrated invasion inhibition by 15–20% at a relatively high concentration of 2 mg/mL (Fig. 7B). Rabbit polyclonal IgG raised to the mammalian recP12- and recP41-Cd4d3/4-6H fusion proteins were similarly ineffective at inhibiting invasion of 3D7 and Dd2 parasite lines (Fig. 7C). While these antibodies were able to block the P12/P41 interaction, they failed to substantially disrupt the heterodimer once formed (Fig. S4).

## Discussion

Because 6-cys family members play important roles in gamete fertilization and hepatocyte infection, we hypothesised that the merozoite 6-cys proteins P12 and P41 may play a similarly important role in merozoite invasion. While we demonstrate that these proteins form a stable heterodimer and are localised to both the merozoite surface and apical region, and so are appropriately positioned for a role in invasion, several lines of evidence fail to demonstrate any significant role for these proteins in this capacity.

Firstly, we examined the role of P12 and P41 in invasion by performing erythrocyte-binding assays. Despite adopting several experimental approaches we failed to detect erythrocyte binding using native heterodimer present in culture supernatant. Also, in using the mammalian-expressed recombinant P12/P41 heterodimer we did not detect specific binding of the heterodimer to any parasite or erythrocyte protein in lysates of whole infected schizonts (data not shown). We found the absence of a detectable binding partner surprising since proteins related to the 6-cys family, namely the homo-dimeric SAG proteins from *Toxoplasma gondii*, are predicted to bind sugars in a groove formed between the paired N-terminal 6-cys domains [44]. The crystal structures of various SAG-like proteins have now been solved and reveal that the N-terminal grooves are heterogeneous in shape and charge indicating their carbohydrate ligands may be quite diverse [45]–[47]. Until very recently the only structures available for 6-cys were modelled on the crystal structure of the SAG1 protein [44]. Arredondo et al. have now solved the NMR structure of the membrane proximal 6-cys domain of P12 in *P. falciparum* and confirmed the previous predicted structure [5]. A structure for full-length recombinant P12 protein could not be derived, however our production of highly pure recP12 and recP41 capable of heterodimerising now offers the prospect of solving the whole structure of a *Plasmodium* 6-cys heterodimer.

Next, we attempted to establish if antibodies to P12 and P41 were capable of inhibiting merozoite invasion of erythrocytes which would serve to validate the potential of these proteins as vaccine targets. At a relatively high IgG concentration of 2 mg/mL, weak invasion inhibitory activity of 10–20% was observed for both antibodies raised to *E. coli* expressed P12 and P41 relative to pre-immune IgG. At lower antibody concentrations invasion inhibitory activity was far less pronounced (data not shown). Moreover, combining the antibodies to both antigens offered no synergistic activity greater than the additive contributions of each individual antibody (data not shown). By comparison, the invasion inhibitory activity of polyclonal antibodies towards AMA1 is quite potent, (up to 50% at 0.5 mg/mL) possibly interfering with AMA1 binding to RON2 ablating successful tight junction formation [48], [49].

The same level of poor invasion inhibitory activity was also evident with antibodies raised to the P12 and P41 fusion proteins expressed in mammalian cells. Based on their ability to form discrete heterodimers, it is likely that these fusion proteins are in their correct conformation and should generate antibodies to native epitopes (Fig. 3A). Antibodies to the mammalian expressed P12 and P41 fusion proteins were effective at preventing heterodimer formation if incubated with their respective monomer prior to co-incubation of the two proteins (Fig. S4). These antibodies were however incapable of disrupting the already formed heterodimer. This suggests that antibody-mediated disruption of the heterodimer is unlikely to occur *in vivo*. We interpret the low level of invasion inhibition observed with anti-P12 and anti-P41 IgGs raised against protein expressed in *E. coli* is non-specific, possibly due to coating of the merozoite surface rather than the inhibition of a specific invasion related function.

Thirdly, we undertook a genetic approach to shed light on the function of P12 and P41 by deleting their genes and assaying for changes in growth rates and invasion pathways. It has been established that deletion of certain parasite invasion ligands can trigger a compensatory increase in the expression of alternative invasion ligands coupled by a switch in invasion pathways. For example, deletion of EBA175 in the W2mef strain leads to the up-regulation of reticulocyte binding homolog 4 (Rh4) expression and a subsequent switch from a sialic acid dependent to a sialic acid independent invasion pathway [50]. Such changes in the parasite's invasion pathway can be detected by measuring differences in the rate of invasion into enzymatically treated erythrocytes. This results in the removal of specific erythrocyte receptors used by the parasite for a given invasion pathway. To assess whether a similar scenario was occurring with respect to Δ*p41*, its growth was compared with the CS2 parental line in trypsin-, chymotrypsin- and sialidase-treated erythrocytes. Over the assayed growth period, no substantial difference in parasite amplification was detected between the Δ*p41* and CS2 (data not shown), suggesting that no significant change in invasion phenotype occurs in the absence of P41. Furthermore, we could not detect any changes in growth rates of either *Δp12* or Δ*p41* parasites *in vitro* (Fig. 7). This was somewhat surprising given the extensive phylogenetic conservation of P41 and P12 orthologs across the *Plasmodium* genus coupled with the aforementioned observations by others that deletion of 6-cys proteins expressed at other life stages leads to dramatic phenotypic changes. Deletion of *p47*, *p48/45* and *p230* in *Plasmodium berghei* leads to dramatic declines in gamete fertilization [19]. In *P. bergehi* and *P. falciparum* deletion of *p36* and *p36p* leads to growth arrest during hepatocytic development even though the sporozoites appear to invade hepatocytes normally [20], [21], [24]. Admittedly though, not all deleted 6-cys genes produce detectable phenotypes since the asexual stage 6-cys member, *p38*, was successfully deleted in *P. berghei* without apparent growth changes [19].

It is interesting to note that some P41 remains detectable in samples prepared from saponin-lysed Δ*p12* parasites despite the loss of its membrane anchored binding partner (Fig. 6B). Although some P41 is also found in the parasitophorous vacuole fraction of Δ*p12* parasite, we can only assume the P41 that remains on the parasite surface must be interacting with other merozoite surface proteins. It would be interesting to identify these other partners even though our initial attempt here to identify these partners was unsuccessful.

Perhaps one reason why a phenotype was not observed for the blood-stage expressed 6-cys null mutants resides in the extensive time these mutants have to adapt *in vitro*. Months of culturing are required to establish such mutants and in this time the completion of 30–40 blood-stage cycles may very well allow for compensatory changes which could serve to restore normal growth. This circumstance is in stark contrast to the deletion mutants of 6-cys proteins expressed at other life stages which are examined phenotypically in the first cycle of that particular stage. In this regard it is interesting to note that while P36 and P36P knockout lines display dramatic phenotypes in hepatocyte invasion, in both cases breakthrough parasites are observed even in this single cycle. Hence, the phenotype is not completely penetrant and it may stand to reason that multiple passages of breakthrough parasites through hepatocytes results in a strongly adapted P36 and P36P knockout line having no discernable hepatocyte development phenotype from the wildtype line. This hypothesis though remains conjecture and ultimately conditional knockdown of *p12* and *p41* expression is needed to examine whether phenotypes can be observed in the first blood-stage cycle.

The fact that the majority of merozoite surface proteins have no known function despite decades of study indicates the difficulties associated with this area of research. MSP1 is the merozoite surface protein for which we have the most advanced functional knowledge, namely the native protein has been proposed to bind to heparin-like molecules on erythrocyte surface [41]. A fragment of MSP1, MSP1_33_, was also recently suggested to have an immunomodulatory role in the human host [51]. The MSP3 null mutant displays reduced invasion rates but this has provided little information on the precise role of this protein [52]. Genetic deletion of other MSP genes has been successful but again has provided little functional information with the parasites exhibiting a modest or no growth delay [53]–[55]. Of those MSPs that appear to be refractory to genetic deletion, this simply indicates they are probably essential for blood-stage growth but provides very little information on their possible functions either [54].

Given the involvement of other 6-cys family members in processes of recognition and adhesion, the possibility remains that the P12/P41 complex plays a non-essential role in *in vitro* culture. Such a role would be difficult to detect by the approaches reported herein and perhaps requires new techniques to capture a low-affinity and presumably transient interaction between the P12/P41 complex and its putative erythrocyte receptors possibly in their native membrane bound forms. It is also possible that P12 and P41 bind to erythrocyte types other than the O-type normocytes used in these experiments and it would be worthwhile in the future repeating these experiments in other blood types especially reticulocytes which support rapid parasite growth [56]. Alternatively, the P12/P41 protein complex may bind to host proteins to facilitate host immunomodulatory roles, functions not detected by our *in vitro* approaches. Given that P12 and P41 are apparently exclusively expressed in the blood-stage, and are evolutionarily conserved throughout the *Plasmodium* genus, they would seem likely to play an important biological function. The fact that this function was not revealed by any of the biochemical or functional assays described here is perhaps indicative of the ultimate limitations of working with *P. falciparum* in an *in vitro* model culture system. However, this work has clearly established that these proteins are likely to function as a heterodimeric complex, and therefore future functional studies should focus on this form.

## Supporting Information

Figure S1 **Crosslinking experiment confirms the native P12/P41 heterodimer formation.** Proteins from magnet-purified schizonts were crosslinked with various concentrations of DSP since achieving optimal crosslinking is empirical. Following schizont lysate extraction, crosslinked proteins were separated by SDS-PAGE and underwent western blot analysis. When probed for P12, a species of approximately 70 kDa was detected in the crosslinked samples corresponding to a predicted single P12/P41 heterodimer in CS2 parasites (upper panel). This heterodimeric species was not detected in the absence of DSP whereas monomeric P12 could be detected in all the samples. When probed with anti-P41 two bands were detected in the 70 kDa range (lower panel). To establish which species corresponded to the P12/P41 dimer, Δ*p41* parasites in a CS2 background were also crosslinked and following SDS-PAGE were western blotted and probed with anti-P41 antibodies. The smaller of the two ∼70 kDa bands detected by anti-P41 antibodies in the parental CS2 line disappeared in the Δ*p41* parasites indicating this band represented the heterodimer.
(TIF)

Figure S2 **Identification of binding partners of P12/P41 complex.** Crosslinked and non-crosslinked schizont lysates of CS2 and Δ*p41* CS2 parasites were immunoprecipitated with rabbit anti-P12 and anti-P41 antibodies immobilised on Protein G beads. Prior to fractionation by SDS-PAGE DSP crosslinks were reduced by treatment with dithiothreitol and folllowing electrophoresis bands unique to the crosslinked sample were excised from the colloidal Coomassie-stained polyacrylamide gels. Proteins were identified by LC-MS/MS sequencing. Bands excised contained the highly abundant merozoite surface/parasitophorous vacuole proteins MSP1, MSP9 and SERA5.
(TIF)

Figure S3 **Southern blotting and PCR show the successful disruption of** ***p12*** **and** ***p41*** **genes.** (A) Genomic DNA of wildtype 3D7, Δ*p12* 3D7, and Δ*p41* 3D7 were extracted and Southern blots performed as outlined in the experimental procedures. Probing with both 5′ flank and 3′ flank, DNA from Δ*p12* and Δ*p41* parasites following 2 rounds (2cyc+5-FC) and 3 rounds (3cyc+5-FC) of drug cycling revealed DNA fragments of expected sizes corresponding to parasites that had undergone double crossover recombination. This is indicative of the deletion of *p12* and *p41*. P41-null parasites were found to be of a mixed population including a subpopulation of wildtype parasites. Consequently Δ*p41* clones were achieved by limiting dilution. (B) The extracted gDNA from 3D7, Δ*p12* clone 1, and Δ*p41* clone 1 was also used to confirm homologous recombination by PCR with double crossover specific primers. All primer pairs yielded expected results for either 5′ or 3′ integration sites of P12-null and P41-null parasites.
(TIF)

Figure S4 **The anti-recP12 and anti-recP41 antibodies are capable of blocking the P12/P41 interaction but cannot disrupt the heterodimer once formed.** The interaction between recP12 and recP41 was detected using the AVEXIS method. (A) The pentameric β-lactamase-tagged recP12 prey was incubated in the presence of increasing concentration of anti-recP12 antibody before being presented to the monomeric biotinylated recP41 bait immobilised on a streptavidin-coated microtitre plate. The interaction was detected by hydrolysis of the β-lactamase substrate nitrocefin forming a product that absorbs at 485 nm. (B) The reciprocal experiment in which the anti-recP41 antibody was pre-incubated with the recP41 prey could also block binding to the recP12 bait. (C) Neither anti-recP12 nor anti-recP41 had the capacity to destabilise the formed recP12-recP41 complex. recP12 (red) or recP41 prey (blue) was presented to its respective bait, prior to the addition of varying concentration of anti-recP12 or anti-recP41, respectively to the formed complex. No decrease in absorbance was observed. All experiments were performed in triplicate; error bar = SD.
(TIF)

Table S1 **Primers used to generate knockout plasmids.**
(DOC)

Table S2 **Primers used to validate homologous recombination in the knockout parasites by PCR.**
(DOC)
