# Supplementary figures and images for: Biochemical and Functional Analysis of Two Plasmodium falciparum Blood-Stage 6-Cys Proteins: P12 and P41
Source: PLoS One. 2012 Jul 27;7(7):e41937. doi: 10.1371/journal.pone.0041937 (PMC3407074; doi:10.1371/journal.pone.0041937)

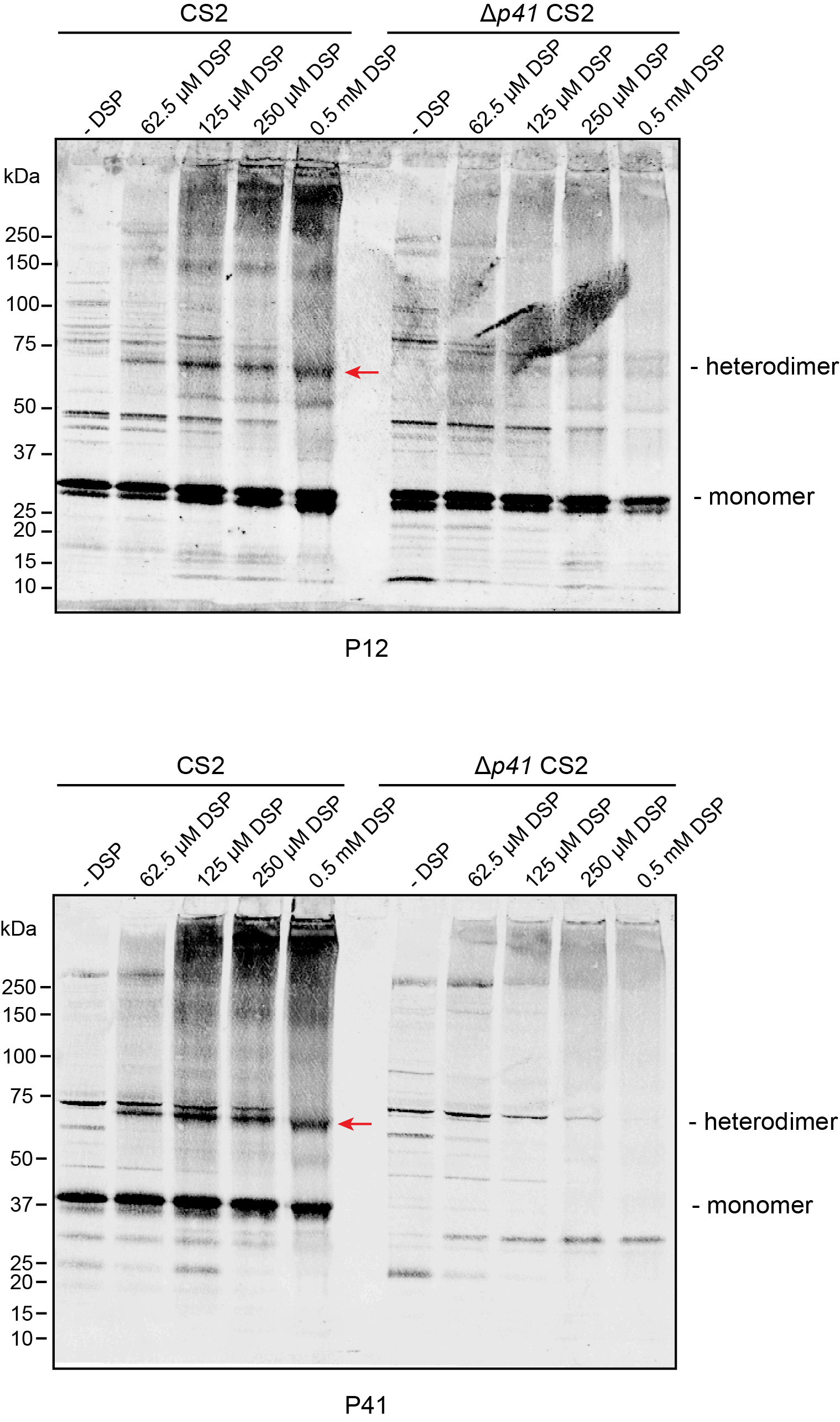

Supplement: Figure S1 — Crosslinking experiment confirms the native P12/P41 heterodimer formation. Proteins from magnet-purified schizonts were crosslinked with various concentrations of DSP since achieving optimal crosslinking is empirical. Following schizont lysate extraction, crosslinked proteins were separated by SDS-PAGE and underwent western blot analysis. When probed for P12, a species of approximately 70 kDa was detected in the crosslinked samples corresponding to a predicted single P12/P41 heterodimer in CS2 parasites (upper panel). This heterodimeric species was not detected in the absence of DSP whereas monomeric P12 could be detected in all the samples. When probed with anti-P41 two bands were detected in the 70 kDa range (lower panel). To establish which species corresponded to the P12/P41 dimer, Δp41 parasites in a CS2 background were also crosslinked and following SDS-PAGE were western blotted and probed with anti-P41 antibodies. The smaller of the two ∼70 kDa bands detected by anti-P41 antibodies in the parental CS2 line disappeared in the Δp41 parasites indicating this band represented the heterodimer. (TIF) [file pone.0041937.s001.tif]

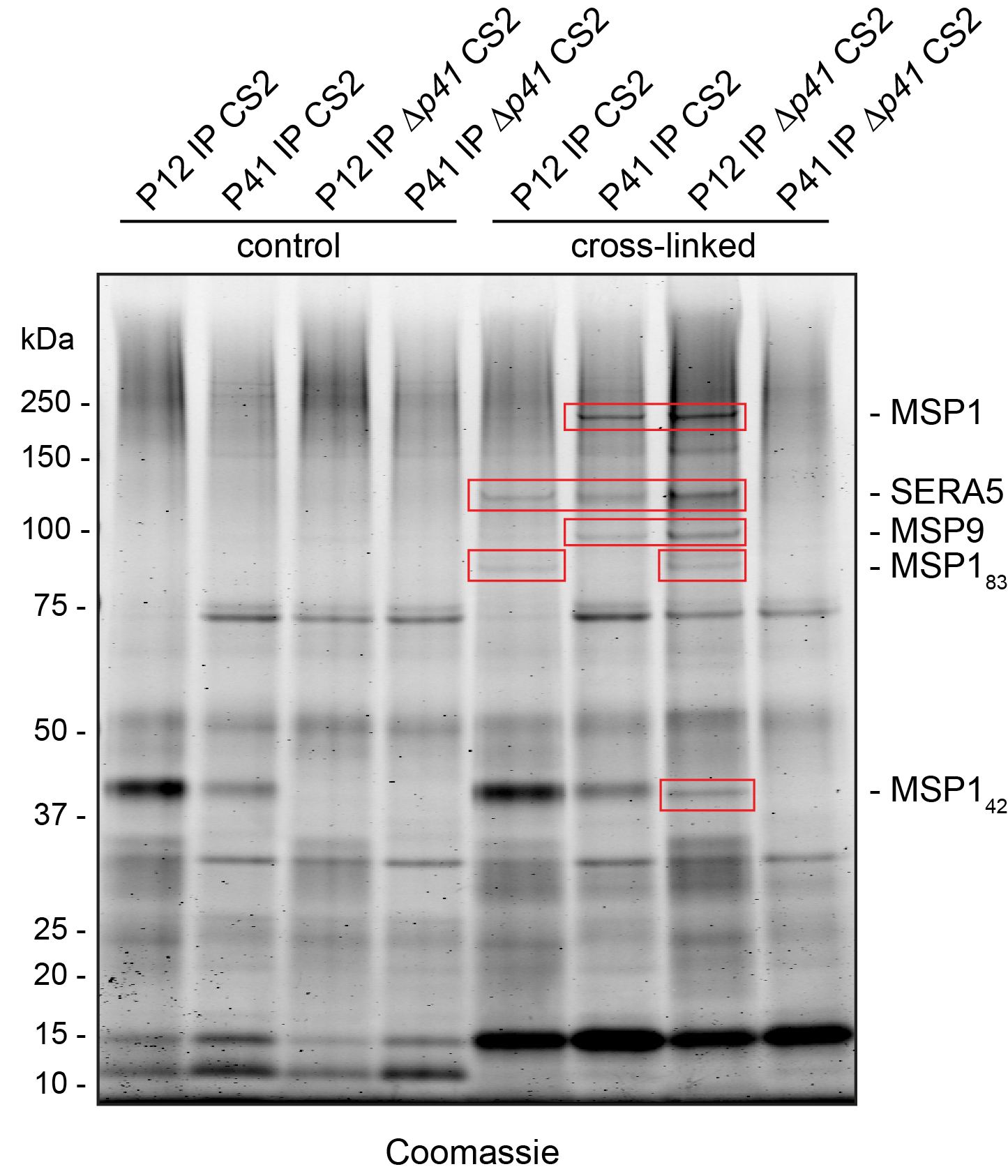

Supplement: Figure S2 — Identification of binding partners of P12/P41 complex. Crosslinked and non-crosslinked schizont lysates of CS2 and Δp41 CS2 parasites were immunoprecipitated with rabbit anti-P12 and anti-P41 antibodies immobilised on Protein G beads. Prior to fractionation by SDS-PAGE DSP crosslinks were reduced by treatment with dithiothreitol and folllowing electrophoresis bands unique to the crosslinked sample were excised from the colloidal Coomassie-stained polyacrylamide gels. Proteins were identified by LC-MS/MS sequencing. Bands excised contained the highly abundant merozoite surface/parasitophorous vacuole proteins MSP1, MSP9 and SERA5. (TIF) [file pone.0041937.s002.tif]

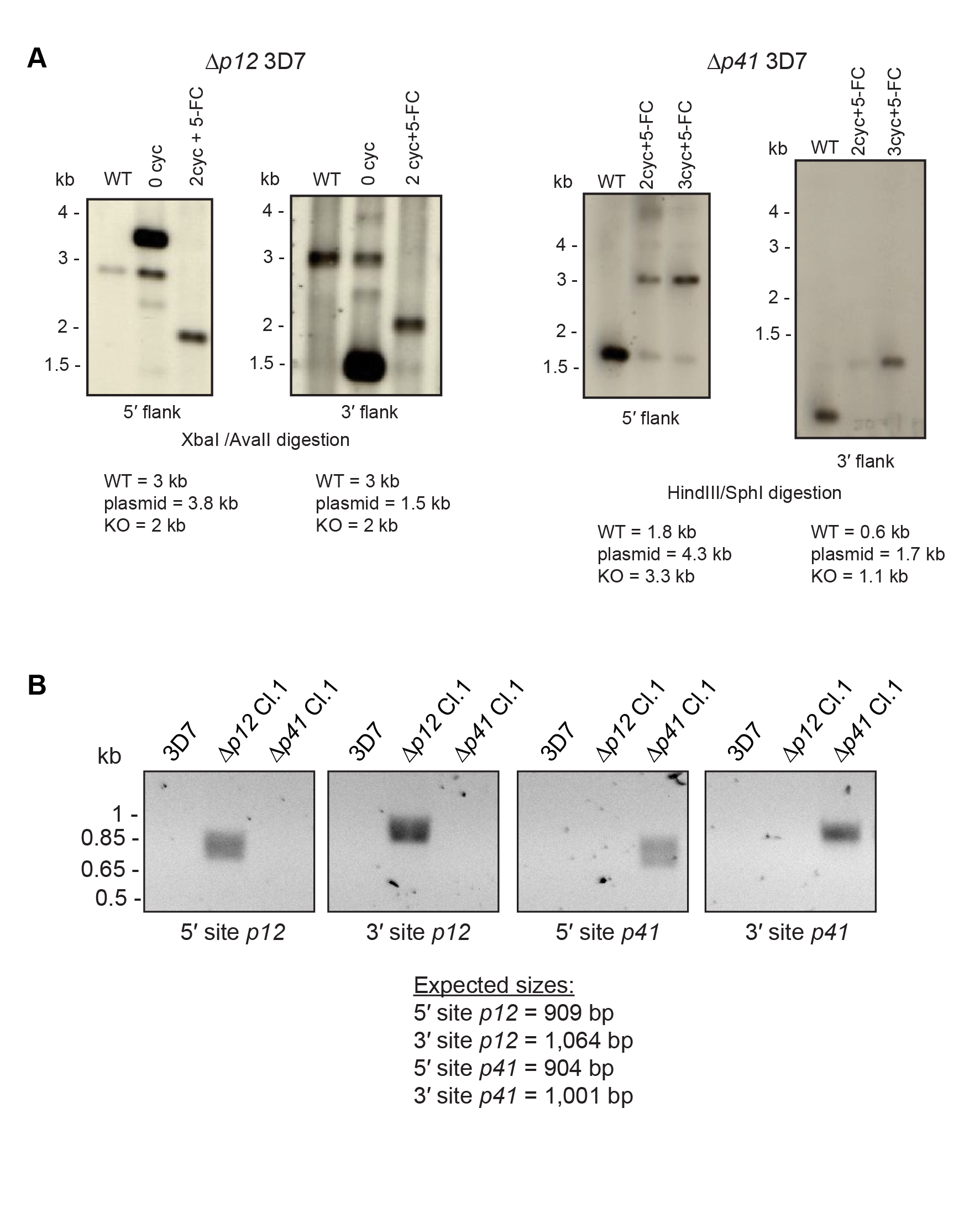

Supplement: Figure S3 — Southern blotting and PCR show the successful disruption of p12 and p41 genes. (A) Genomic DNA of wildtype 3D7, Δp12 3D7, and Δp41 3D7 were extracted and Southern blots performed as outlined in the experimental procedures. Probing with both 5′ flank and 3′ flank, DNA from Δp12 and Δp41 parasites following 2 rounds (2cyc+5-FC) and 3 rounds (3cyc+5-FC) of drug cycling revealed DNA fragments of expected sizes corresponding to parasites that had undergone double crossover recombination. This is indicative of the deletion of p12 and p41. P41-null parasites were found to be of a mixed population including a subpopulation of wildtype parasites. Consequently Δp41 clones were achieved by limiting dilution. (B) The extracted gDNA from 3D7, Δp12 clone 1, and Δp41 clone 1 was also used to confirm homologous recombination by PCR with double crossover specific primers. All primer pairs yielded expected results for either 5′ or 3′ integration sites of P12-null and P41-null parasites. (TIF) [file pone.0041937.s003.tif]

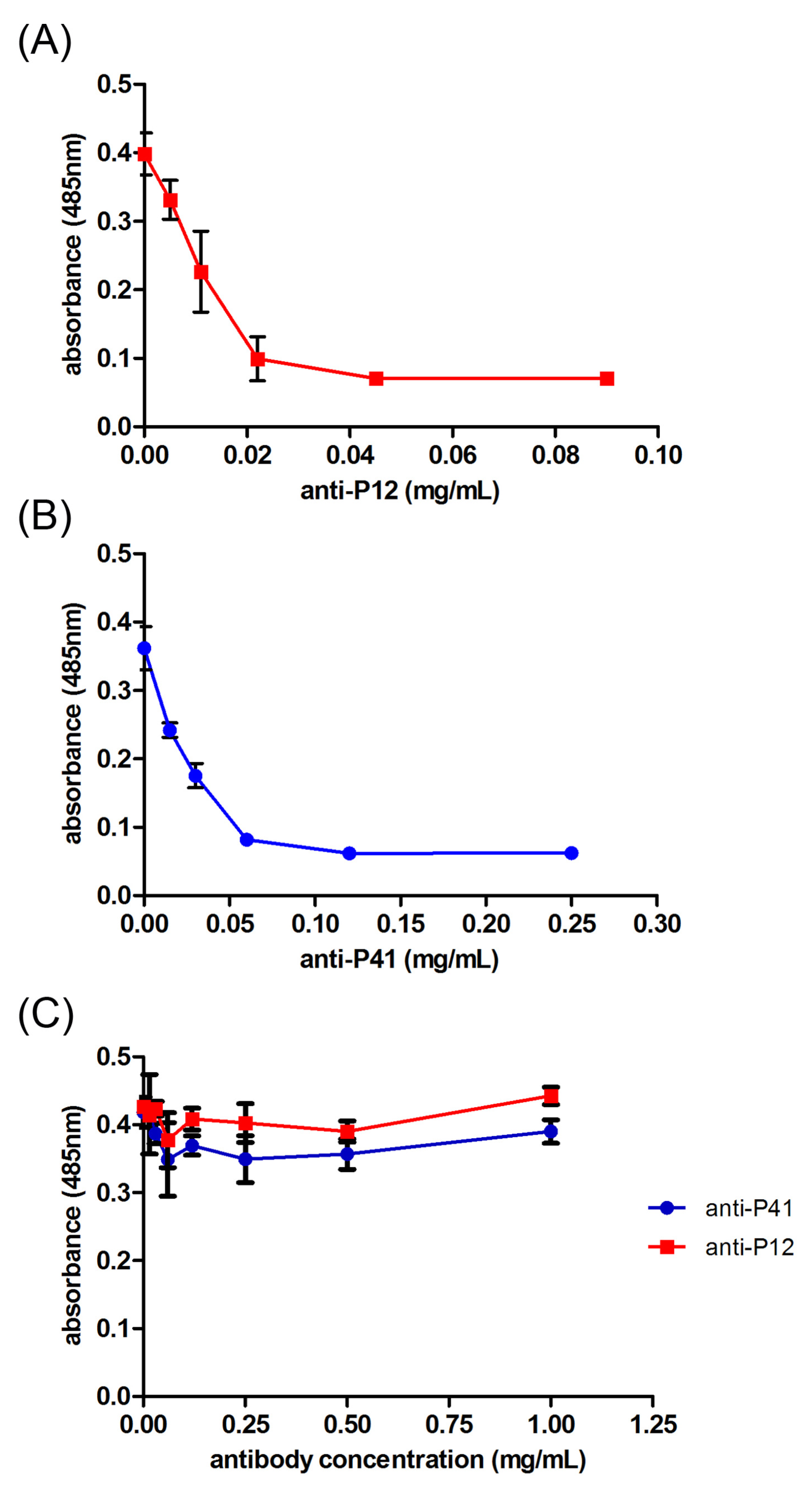

Supplement: Figure S4 — The anti-recP12 and anti-recP41 antibodies are capable of blocking the P12/P41 interaction but cannot disrupt the heterodimer once formed. The interaction between recP12 and recP41 was detected using the AVEXIS method. (A) The pentameric β-lactamase-tagged recP12 prey was incubated in the presence of increasing concentration of anti-recP12 antibody before being presented to the monomeric biotinylated recP41 bait immobilised on a streptavidin-coated microtitre plate. The interaction was detected by hydrolysis of the β-lactamase substrate nitrocefin forming a product that absorbs at 485 nm. (B) The reciprocal experiment in which the anti-recP41 antibody was pre-incubated with the recP41 prey could also block binding to the recP12 bait. (C) Neither anti-recP12 nor anti-recP41 had the capacity to destabilise the formed recP12-recP41 complex. recP12 (red) or recP41 prey (blue) was presented to its respective bait, prior to the addition of varying concentration of anti-recP12 or anti-recP41, respectively to the formed complex. No decrease in absorbance was observed. All experiments were performed in triplicate; error bar = SD. (TIF) [file pone.0041937.s004.tif]
