# Supplementary material for: Biochemical and Functional Analysis of Two Plasmodium falciparum Blood-Stage 6-Cys Proteins: P12 and P41
Source: PLoS One. 2012 Jul 27;7(7):e41937. doi: 10.1371/journal.pone.0041937 (PMC3407074; doi:10.1371/journal.pone.0041937)
Supplement: Table S1 — Primers used to generate knockout plasmids. (DOC) [file pone.0041937.s005.doc]

| **Gene regions** | **Primers** | **Sequences (5' - 3')** |
| --- | --- | --- |
| **5' *p12* (3D7)** | Forward | ATCCCGCGGCCACCCACATGTGGTGTG |
|  | Reverse | GATACTAGTCTCATAAACATTGATGATCCG |
| **3' *p12* (3D7)** | Forward | ATCGAATTCTTACAACAAGCGAATCTAC |
|  | Reverse | GATCCTAGGTTGAGTTGTTTCAAAGTCC |
| **5' *p41* (3D7)** | Forward | AATCCGCGGGTCACATATACATATAAATGTGTATATATATC |
|  | Reverse | ATTACTAGTCATCGTCATCTTTTAGGAACCTATTG |
| **3' *p41* (3D7)** | Forward | ATTGAATTCCAAATGATATATTAAACTATGATG |
|  | Reverse | CAACCTAGGCTTCACTTTCTCCAGGTTGTATATC |
| **5' *p41* (CS2)** | Forward | ATCCCGCGGTGTGATGCCAAATTTACAG |
|  | Reverse | GATACTAGTCACAAGATACTTCCTTCTCTCC |
| **3' *p41* (CS2)** | Forward | ATCGAATTCGTAATCTGGACACGGTGACAATAC |
|  | Reverse | GATCCTAGGAAATCCAACTAATTCTCCAGG |
