# Supplementary material for: Biochemical and Functional Analysis of Two Plasmodium falciparum Blood-Stage 6-Cys Proteins: P12 and P41
Source: PLoS One. 2012 Jul 27;7(7):e41937. doi: 10.1371/journal.pone.0041937 (PMC3407074; doi:10.1371/journal.pone.0041937)
Supplement: Table S2 — Primers used to validate homologous recombination in the knockout parasites by PCR. (DOC) [file pone.0041937.s006.doc]

| **Sites** | **Primers** | **Sequences (5' - 3')** |
| --- | --- | --- |
| **5' *p12*** | Forward | TAGTGGAAAGGACAATATAAAGGG |
|  | Reverse | GCTTAAGACAGATCTTCGGACTAG |
| **3' *p12*** | Forward | GGGATAGCGATTTTTTTTACTGTC |
|  | Reverse | TATCTGCTATTAAAACTATGAAATTAAACC |
| **5' *p41*** | Forward | TTATGTGTGATGCCAAATTTACAG |
|  | Reverse | GCTTAAGACAGATCTTCGGACTAG |
| **3' *p41*** | Forward | GGGATAGCGATTTTTTTTACTGTC |
|  | Reverse | CTTTTGAAATCTTAATAGTATGCACATAC |
